# Prostatic biopsy-related rectal bleeding refractory to medical and endoscopic therapy definitively managed by catheter-directed embolotherapy: a case report

**DOI:** 10.1186/s13256-015-0727-0

**Published:** 2015-10-29

**Authors:** Tom De Beule, Kenneth Carels, Sabine Tejpar, Ben Van Cleynenbreugel, Raymond Oyen, Geert Maleux

**Affiliations:** 1 Department of Radiology, University Hospitals Leuven, Herestraat 49, B-3000 Leuven, Belgium; 2 Department of Imaging and Pathology, KU Leuven, Leuven, Belgium; 3 Department of Gastroenterology, University Hospitals Leuven, Leuven, Belgium; 4 Department of Urology, University Hospitals Leuven, Leuven, Belgium

**Keywords:** Embolotherapy, Prostatic biopsy, Rectal bleeding

## Abstract

**Introduction:**

Ultrasound-guided transrectal prostatic biopsy is generally a well-tolerated radiological technique with low overall complication ratio. If post-biopsy rectal bleeding occurs, conservative management is effective in the majority of cases. Endoscopic or interventional treatment is rarely required.

**Case presentation:**

We report the case of an 82-year-old white man presenting with massive rectal bleeding after ultrasound-guided prostatic biopsy. Medical and endoscopic management were not effective. Angiographic evaluation revealed a prostatic arteriovenous fistula, and definitive treatment was provided in the form of catheter-directed superselective embolotherapy.

**Conclusion:**

Transrectal prostatic biopsy may be associated with massive rectal bleeding. Transcatheter embolotherapy can be effective in definitively stopping the bleeding.

## Introduction

Ultrasound (US)-guided transrectal prostatic biopsy is commonly performed for detection and diagnosis of prostate carcinoma. In the majority of cases this procedure is associated with small numbers of minor complications ranging from self-limiting rectal bleeding and hemospermia to urinary symptoms, not usually requiring additional intervention [1]. Post-biopsy rectal bleeding is uncommon with an incidence of approximately 2.5 % [2], but can be life-threatening in very rare cases. Common treatment options are balloon tamponade, endoscopic adrenaline injection and endoclipping [3–5]. In our case endoscopic management failed and catheter-directed embolotherapy was performed to control the rectal hemorrhage.

## Case presentation

**Fig. 1:**
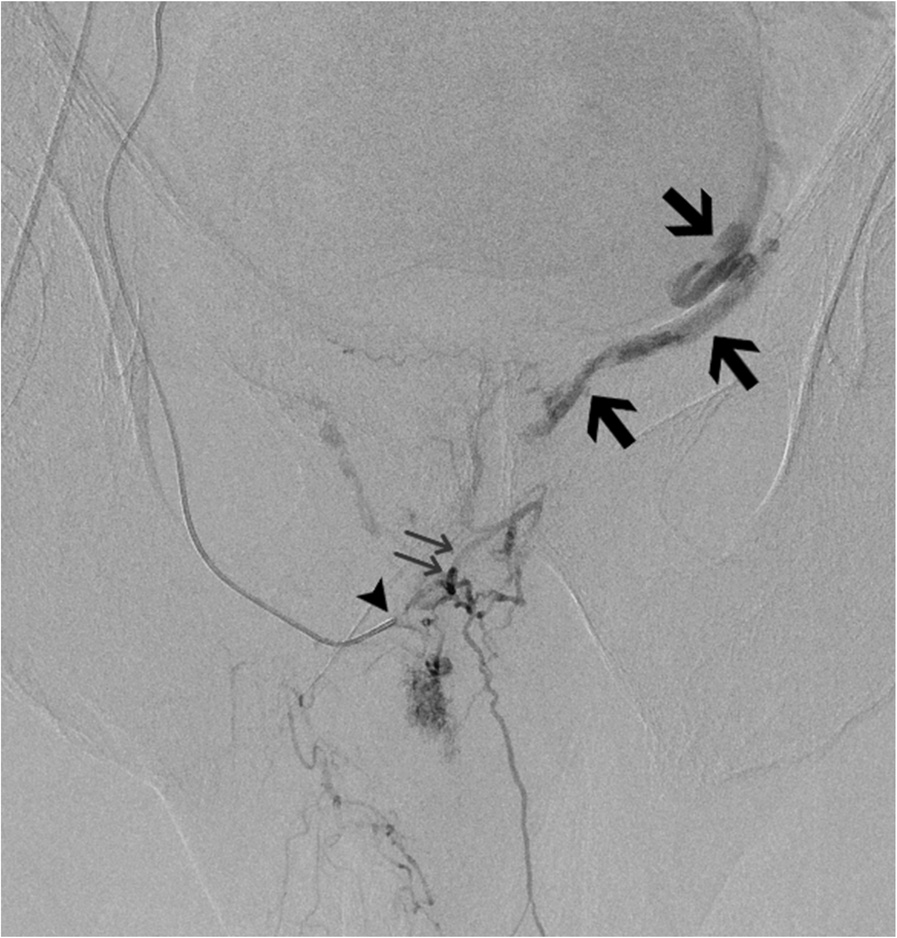
Contrast injection through a microcatheter (*arrowhead*) with its tip in the internal pudendal artery: opacification of left prostatic gland through an anastomosis from the internal pudendal artery and the left inferior prostatic artery (*arrows*). Note also the arteriovenous fistula with a large draining vein (*large arrows*) in the left prostatic site after selective catheterization with opacification of the dorsal penile artery

**Fig. 2:**
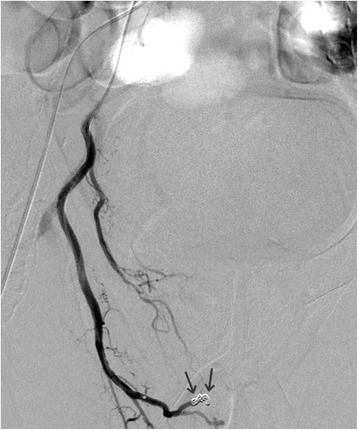
Control angiography after embolization with microparticles (300–500 μ) and proximal microcoil (*arrows*) occlusion of the anastomosis with the left inferior prostatic artery

**Fig. 3:**
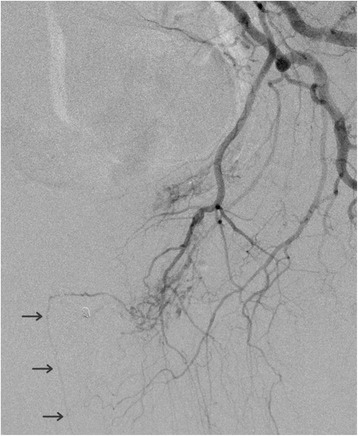
Selective angiography of the left internal iliac artery shows normal opacification of the dorsal penile artery (*arrows*) originating from the left internal pudendal artery. Absence of opacification of an arteriovenous fistula

An 82-year-old white man with a medical history of paroxysmal atrial fibrillation for which coumarins were prescribed presented with an episode of macroscopic hematuria. A physical rectal examination revealed a diffusely indurated prostate and computed tomography (CT) showed normal aspect of his kidneys and bladder as well as an enlarged prostate. Laboratory analysis showed a white blood cell count within normal limits (6.69×10^9^/L) and a slight increase in prostate-specific antigen from 4 to 9.4 ng/ml. A transrectal US of his prostate revealed a hypoechoic and hypervascular subcapsular area in the peripheral zone of his prostate with a differential diagnosis including diffuse carcinoma or granulomatous inflammation. An uneventful US-guided transrectal biopsy was performed on an out-patient basis, 7 days after anticoagulation therapy was ceased. Pathologic analysis of the biopsy specimen revealed a diffuse carcinoma of the peripheral zone of his prostate. Three days later, a massive rectal hemorrhage occurred, associated with hemodynamic shock (blood pressure 50/30 mmHg, heart rate 100 beats per minute). A good hemodynamic response was obtained after appropriate therapeutic management. A clinical examination revealed a nodular rectal area in the prostate bed without a large hematoma. Endoscopy revealed two active arterial bleeders in his lower rectum but endoscopic clipping failed. He was referred to the angiography suite for emergency interventional treatment. Selective angiography of his inferior mesenteric artery did not reveal any bleeding. Selective catheterization of the anterior division of his right internal iliac artery revealed a prostatic artery with an inferior and superior branch. With a microcatheter (Progreat 2.7, Terumo Europe, Leuven, Belgium), selective catheterization of his internal pudendal artery revealed an arteriovenous fistula (AVF) in the left prostatic body, fed from collaterals originating from his right inferior prostatic artery (Fig. 1). No contrast extravasation was noted. Embolization with calibrated microparticles (Embosphere® 300–500 μ, Merit Medical, South Jordan, Utah, USA) was performed, followed by placement of three 4×4 mm microcoils (Target®, Boston Scientific Inc Natick, MA, USA) at the origin of the anastomosis with his left inferior prostatic artery (Fig. 2). Control angiography following embolization showed complete occlusion of the treated artery without residual opacification of the AVF. Selective angiography of his left internal iliac artery showed a patent internal pudendal artery with normal opacification of his dorsal penile artery and without opacification of an AVF (Fig. 3). He was discharged the next day without clinical signs of postembolization syndrome or lower urinary tract symptoms.

## Discussion

Prostatic biopsy has become a routine procedure for cancer detection with low risk for complications. Out of all patients, 70 to 92 % have little or no discomfort [1] ranging from hematuria and hematospermia to minor rectal bleeding and lower urinary tract syndrome. Major complications are rare. Severe rectal bleeding occurring within 2 days is known to be one of the major causes of hospital readmission (0.3 to 2.5 %). Risk factors for major rectal hemorrhage do not seem to be influenced by the use of aspirin or coumarins [1]. Increased sample size and increased number of biopsy cores are correlated with increased bleeding risk, starting from 16 gauge needles and 10 cores [6]. Most rectal hemorrhages are self-limiting if they occur in the first 2 days after biopsy, and do not require treatment. The use of manual, balloon or US-guided compression [7] and endoscopic clipping is successful in a large majority of cases [4].

In this case, a prostatic AVF was found after biopsy and associated with massive rectal bleeding requiring readmission on day 2. Prostatic AVF after biopsy may occur in 13 % of benign prostatic lesions and 90 % of these are transient with spontaneous closure within 18 minutes. None are associated with major bleeding. Biopsy of malignant prostatic lesions, however, tends to create a transient AVF in up to 40 % of cases; this is probably due to increased neovascularity [8]. A complicated AVF with rectal hemorrhage requiring interventional radiological treatment is seldom seen and to the best of our knowledge has not yet been described in the literature.

In the case presented here, the prostatic vascular supply originated from the anterior division of the internal iliac artery branching from the pudendal artery and resulting in a prostatic artery with superior and inferior branches. This type of prostatic vascular supply is common, although there is wide anatomical variation [9]. Alternative origins of the prostatic artery occur in almost 60 % of cases. The most common variants originate from the superior vesical artery (20 %) or the anterior common gluteal-pudendal trunk (18 %). Knowledge of these anatomical differences is crucial when performing an embolization in the vascular territory of the prostate. In particular, attention has to be paid to the large incidence of prostatic anastomosis with the internal pudendal arteries (43 %), contralateral arteries and sometimes the rectal arteries (14 %).

In our case the AVF was visible in the left lobe after contrast injection in the right prostatic artery due to the vascular anastomoses between the right and left sides of the prostate (Fig. 2). Two types of anastomoses are described by Bilhim et al. [10]: a small pericapsular anastomosis or the large precapsular anastomoses which are visible on angiographic examination. In 3.3 % of cases the blood supply to the corpora cavernosa originates from the anastomosis with the internal pudendal artery originating from the prostatic artery.

Embolotherapy was performed through a superselective approach. Particulates in combination with microcoils were used as embolic agents: microparticles occluded the distal small collaterals and a few microcoils were placed more proximally. The bilateral blood supply to the corpora cavernosa was noted before embolization of the right internal pudendal artery side-branches. The location of the AVF, as in this case, supports the importance of the anastomotic collaterals in the prostatic gland supplying the left inferior part of the prostate from the internal pudendal artery on the right.

## Conclusion

A unique case of massive rectal bleeding after US-guided transrectal prostatic biopsy is described. Medical and endoscopic management failed. Selective angiography revealed an AVF arising from the collaterals of the right inferior prostatic artery. Definitive treatment was achieved by selective embolization of the inferior branch of the prostatic artery using a combination of microparticles and microcoils.

## Consent

Written informed consent was obtained from the patient for publication of this case report and accompanying images. A copy of the written consent is available for review by the Editor-in-Chief of this journal.
